# Expert consensus on monitoring antimicrobial stewardship in French nursing homes using assessed reimbursement database indicators

**DOI:** 10.1093/jacamr/dlad037

**Published:** 2023-03-31

**Authors:** Antoine Asquier-Khati, Colin Deschanvres, Anicet Chaslerie, Ouarda Pereira, David Boutoille, Gabriel Birgand

**Affiliations:** Infectious Disease Department, Hotel-Dieu University Hospital, 1 Pl. Alexis Ricordeau, 44093, Nantes, France; Infectious Disease Department, Hotel-Dieu University Hospital, 1 Pl. Alexis Ricordeau, 44093, Nantes, France; Medical Department, Regional Health Insurance Grand Est, Strasbourg, France; Medical Department, Regional Health Insurance Pays de la Loire, Nantes, France; Infectious Disease Department, Hotel-Dieu University Hospital, 1 Pl. Alexis Ricordeau, 44093, Nantes, France; Regional Center for Infection Prevention and Control Pays de la Loire, Hotel-Dieu University Hospital, Nantes, France; National Institute for Health Research Health Protection Research Unit in Healthcare Associated Infection and Antimicrobial Resistance at Imperial College, Hammersmith Campus, London, UK

## Abstract

**Objectives:**

Monitoring the appropriateness of antibiotic prescriptions with indicators based on reimbursement data is required to guide antibiotic stewardship (AMS) interventions in nursing homes (NHs). Quantity metrics (QMs) monitor the volume of prescriptions while proxy indicators (PIs) reflect the appropriateness of antibiotic use. Our objectives were: (i) to provide a relevant consensual set of indicators to be used in French NHs; and (ii) to assess the feasibility of their implementation at the national and local scale.

**Methods:**

Nine French professional organizations implicated in AMS in NHs were asked to nominate at least one member to create a national expert panel of 20 physicians. Twenty-one recently published QMs and 11 PIs were assessed by the expert panel. Indicators were evaluated using a RAND-modified Delphi procedure comprising two online surveys and a videoconference meeting. Indicators were kept in the final list if >70% of stakeholders validated their relevance for estimating the volume (QMs) and appropriateness (PIs) of prescriptions.

**Results:**

Of the 21 QM indicators submitted to the panel, 14 were selected, describing the consumption of antibiotics overall (*n* = 3), broad-spectrum (*n* = 6) and second-line antibiotics (*n* = 2). The three remaining QMs evaluated the route of administration (*n* = 1) and urine culture prescriptions (*n* = 2). Ten PIs (six modified, two rejected, one new) were selected to assess the appropriateness of prescriptions for urinary tract infections (*n* = 2), seasonal variations in prescriptions (*n* = 2), repeated prescriptions of fluoroquinolones (*n *= 1), cephalosporins’ route of administration (*n* = 1), duration of treatment (*n* = 1), rate of second-line antibiotics (*n *= 1), co-prescriptions with non-steroidal anti-inflammatory drugs (*n* = 1), and flu vaccine coverage (*n* = 1). The panel was in favour of using these indicators for regional and facility level AMS programmes (91%), feedback to NH prescribers (82%), benchmarking by health authorities (55%) and public reporting at the facility level (9%).

**Conclusions:**

This consensual list of indicators, covering a wide range of frequent clinical situations, may be used as part of the French national AMS strategy for monitoring antibiotic prescriptions in NHs at the national and local levels. Regional AMS networks might manage this selected list to guide personalized action plans with concrete objectives of reducing the quantity and improving the quality of antibiotic prescriptions.

## Introduction

Antimicrobial resistance (AMR) is recognized as one of the 10 top priority global health issues by the WHO.^1^ Since misuse of antibiotics is one of the main drivers of resistance, antimicrobial stewardship (AMS) aims to promote the responsible use of antimicrobials.^2^ Monitoring antibiotic use (both appropriateness and volume) is essential for guiding antibiotic stewardship programmes.^3^

Nursing home (NH) residents represent a significant and growing part of the population of high-income countries, and are frequently exposed to antibiotics,^3^ especially due to suspected urinary tract infections.^4^ In French NHs, total antibiotic use was around 44 DDDs per 1000 patient-days in 2013,^5^ with at least half of the residents receiving one or more antibiotic prescriptions per year.^6^ While many efforts have targeted hospital settings, NHs remain overlooked in AMS programmes, despite frequent misuse of antibiotics^7^ and high prevalence of antimicrobial-resistant organisms.^8^ In 2019 in France, 8.7% of *Escherichia coli* isolated from urine samples in NH residents were producing ESBLs.^9^

Optimizing antimicrobial use in NHs requires standardized, comparable monitoring and feedback of antibiotic use to guide AMS efforts.^10,11^ Developing indicators that rely on easily available routine data is usually preferred on a large scale for feasibility purposes.^12–15^ A recent study was conducted to design a set of indicators assessing antibiotic use in NHs,^16^ based on the French National Health Data System (SNDS).

In France, each NH’s resident has their own GP, and antibiotic prescriptions are mostly delivered by community-based pharmacy, since >80% of NHs don’t have on-site pharmacy.^16^ Antibiotic reimbursement data are easily available in national databases, giving information about the quantity of antibiotics dispensed by community pharmacies, but without the clinical context of prescription. Two types of indicators are currently used, quantity metrics (QMs) and proxy indicators (PIs). QMs measure the volume of antibiotic delivery and can be easily calculated, but they give poor indication about possible misuse of antibiotics. On the other hand, PIs can reflect the appropriateness of antibiotic prescriptions in the absence of medical data available, and provide concrete targets for improvement.

The aim of the present study was to select relevant indicators, adapt the definition of the QMs and PIs if necessary, and select the targets for PIs using a Delphi process involving a national expert panel. Finally, we assessed the feasibility and explored the potential use of these indicators in a national AMS monitoring programme.

## Material and methods

### Development of a relevant set of indicators for AMS in NHs

#### Initial set of indicators used

As detailed previously,^16^ in France, most NHs (>80%) are community-based, without in-house pharmacies. In such settings, antibiotics are prescribed by GPs, dispensed by community pharmacies, and reimbursed by the National Health Insurance (NHI). Different residents accommodated in the same NH can have different GPs. The indicators used in the present study for AMS in NHs were based on the NHI reimbursement database (SNDS). This database includes the following information for all prescriptions: age and gender of the patient; identification of the prescriber; information on the antibiotic dispensed; and the facility identification in case of NH residency. Information on diagnoses and the duration of treatment in days is not available in the database. However, the number of packages dispensed is collected. The NHI database does not include hospital-based NHs (with on-site antibiotic delivery), representing less than 20% of French NHs. For each indicator, individual prescriptions were aggregated at the NH level. QMs estimated the volume of antibiotic consumption per resident-days, using number of prescriptions and/or DDDs, with a numerator–denominator combination. PIs estimated the appropriateness of antibiotic prescriptions at the NH level and were associated with targets reflecting compliance with national guidelines.

#### Composition of the national expert panel

In November 2020, nine French professional organizations were asked by e-mail to nominate at least one member to take part in the expert panel, favouring people with demonstrated experience and knowledge in the field of AMS in NHs.

#### Study design

A four-step RAND-modified Delphi procedure^17,18^ was used to validate a set of indicators for AMS in NHs. Considering the lack of evidence-based medicine in the field of indicators for AMS, RAND studies can be useful to combine the best available scientific data with the collective statement of experts, in order to yield a consensual list of indicators.^17^ Theses procedures have the advantage to provide: (i) iteration, permitting experts to change their opinion between the different rounds; (ii) controlled feedback, by communicating the results of previous rounds; and (iii) high-quality statistical data, assuring that perspectives of each expert are equally represented in the final set.^19^ Our study comprised two online surveys separated by a videoconference meeting. For the first round of the consensus procedure, we used an initial list of 21 QMs and 11 PIs, recently published by Simon *et al.*,^16^ based on a systematic literature review, including DRIVE-AB (European project Driving Re-InVEstment in R&D and responsible AntiBiotic use) results,^20^ and adapted to the French NH context. This list of indicators was converted into a digital web-based questionnaire using Google Forms^®^ (Data file S1, available as Supplementary data at *JAC-AMR* Online). The expert panel received by e-mail an invitation to complete the online survey, accompanied by an explanatory document (Data file S2) indicating the principles of the indicators, the rationale for the study, the intended objectives, and the scientific references for each of the indicators. For each indicator, experts had to complete a 5-point Likert scale (very poor, poor, fair, good and excellent) reflecting the relevance of the indicator for assessing the volume (QM) and appropriateness (PI) of antibiotic prescriptions respectively. Overall relevance for each indicator was considered fair if the mean score was ≤3.5, good if the mean score was 3.5, good if the mean score was >3.5 and ≤4, and excellent if the mean score was >4. Experts were encouraged to justify their rating, suggest modifications for each component of the indicators if needed (numerator, denominator and target) and, if necessary, suggest new indicators. Experts were not able to reject indicators during this first round. Data were analysed by two independent researchers (A.A.K. and G.B.).

For the second round, experts who participated in the online questionnaire were invited to take part in a 2 h videoconference. During the meeting, the results of the experts’ first-round ratings, with median scores for each indicator and a detailed summary of individual replies, were presented to experts to initiate the discussion. Experts were invited to discuss the ratings, focusing on points of disagreement, and were given the opportunity to modify, remove or add indicators. A consensus between experts was required before proceeding to the next indicator. The discussion was moderated by two external researchers (A.A.K. and G.B.). From the outset, experts were informed that the conference was recorded for audio transcription.

During the third round, a summary report containing all changes validated during the videoconference was sent to the expert panel. Experts were asked to return free-form comments on the report and to suggest final modifications to the indicators, particularly for those that were newly added.

The fourth round relied on a second survey to assess the updated list of indicators. First, for each individual indicator, experts had to state whether they would retain it in the final list or not. Indicators were retained if >70% of experts validated their relevance for estimating the volume (QMs) and appropriateness (PIs) of antibiotic prescriptions, 70% being the threshold usually selected in the literature for RAND studies.^21,22^

### Practical use of selected indicators for the national and local monitoring of antibiotic prescriptions

The final list of indicators was tested using the NHI database in two different French areas for the year 2019. We included all community-based NHs in the Pays de la Loire and Grand Est regions (respectively 3 787  000 and 2 338 000 inhabitants) that were active in 2019. The calculation of QMs and PIs was performed according to the method described elsewhere.^16^

On an exploratory basis, experts were secondly asked to give their opinion regarding four possible uses of the indicators: (i) comparisons between NHs in a benchmarking process; (ii) design of AMS programmes at the facility level; (iii) feedback to NH prescribers; and (iv) public reporting of facility-level indicators.

### Ethics statement

To calculate the set of indicators, we used the National Health Data System (SNDS), a strictly anonymous database, comprising all mandatory national health insurance reimbursement data. No informed consent was required because data were anonymized.

## Results

### Development of a relevant set of indicators for AMS in NHs

A panel of 20 experts from nine different organizations was created for the structured consensus procedure. Of these, five were AMS physicians, four NH physicians, three infectious diseases specialists, two geriatricians, two infection control specialists, two policymakers, one GP and one pharmacist. (Table S1)

#### First online survey

**Figure 1.**
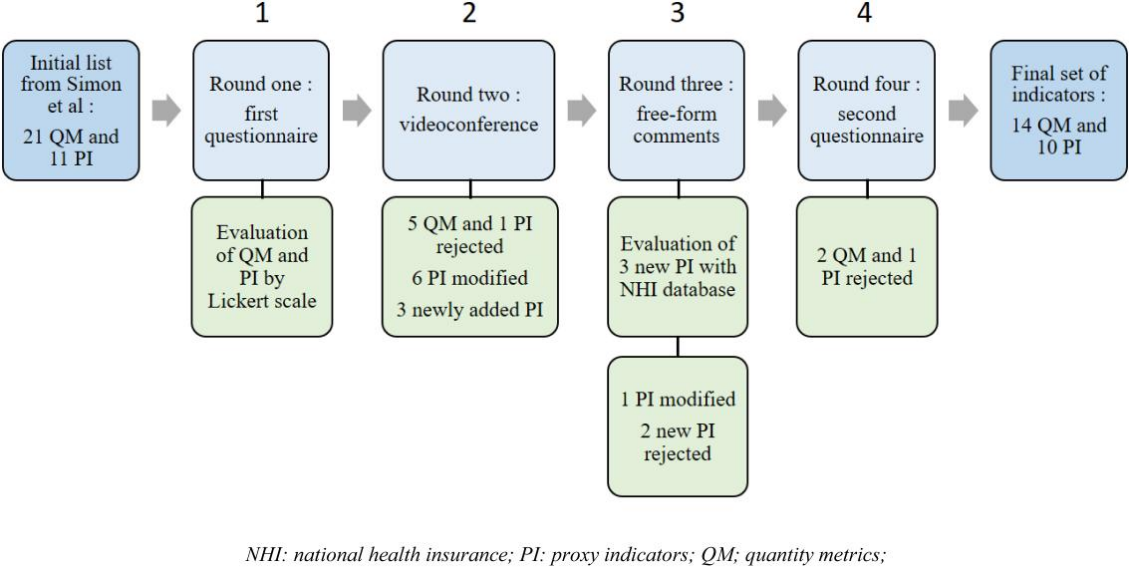
Flow chart of the Delphi consensus procedure.

The first round was conducted from December 2020 to January 2021. Of the 21 potential QMs (Table S2), 7 were evaluated as excellent indicators (mean score > 4). QMs expressed by the absolute number of prescriptions had higher rates than those expressed by the DDD. Of the 11 potential PIs (Table S3), 5 were evaluated as excellent indicators. A flowchart summarizing the results obtained from the four stages in the Delphi procedure is presented in Figure 1.

#### Expert panel meeting

The videoconference meeting took place on 22 February 2021. During this stage, five QMs were rejected from the initial list. The other QMs were retained without modification. Of the PIs, four were kept while six underwent substantial changes to the numerator, denominator or target. Regarding the PI related to the duration of antibiotic prescriptions, the definition of ‘prolonged antibiotic course’ was modified from >8 to >7 days, to comply with recent guidelines and AMS programmes.^23^ Only one PI, related to co-prescriptions of antibiotics with corticosteroid, was removed to consider clinical situations requiring theses associations (i.e. exacerbated COPD). Three additional PIs were suggested by the panel: two PIs related to the route of antibiotic administration (prescriptions for parenteral quinolones and oral cephalosporins) and one PI capturing the switch in antibiotic treatment based on antimicrobial susceptibility testing in urinary tract infections.

#### Review of the summary report by the expert panel

In April 2021, the summary report of the videoconference meeting was sent for review, comments and amendment. After reading the report, experts validated the modification made during the second round: suppression of five QMs and one PI, and the amendments made to six PIs. Another modification was made to one PI during this round. Of the three PIs added in the second round, the experts decided to remove the one related to the prescription of parenteral quinolones due to insufficient room for improvement. The PI reflecting the switch in antibiotic treatment for urinary tract infections was also rejected due to poor clinical meaning.

#### Second online survey

The fourth round was completed between May and June 2021. A total of 27 potential QIs (16 QMs and 11 PIs) were included in the second online questionnaire. Two QMs that did not achieve the 70% positive response rate were rejected, with a mean consensus score of 63.6%. One PI reflecting prescriptions of antibiotics that are not indicated was also rejected, with a score of 63.6%. The mean consensus score for the remaining indicators was 90.3% for the final list of QMs and PIs. The final set of indicators was composed of 14 QMs and 10 Pis, presented in Tables 1 and 2, respectively.

**Table 1.** Final set of 14 quantity metrics after consensus statement with Delphi method, and results calculated at the NH level for two French areas

No.	Consensus	Field	Calculation description	Pays de la Loire area, median (IQR)	Lorraine area, median (IQR)
QM 1	Maintained	Global antibiotic consumption	Number of antibiotic prescriptions (J01)/100 resident-days	0.52 (0.41–0.66)	0.52 (0.42–0.65)
QM 2	Maintained	Global antibiotic consumption	DDDs of antibiotics (J01)/100 resident-days	5.51 (4.25–6.91)	5.34 (4.17–6.78)
QM 3	Maintained	Global antibiotic consumption	Number of residents receiving at least one antibiotic (J01) per year/total number of residents per year	47.89 (41.86–54.65)	52.56 (45.24–59.42)
QM 4	Maintained	Broad-spectrum antibiotics	Number of prescriptions of amoxicillin/clavulanate (J01CR02)/100 resident-days	0.09 (0.07–0.120)	0.08 (0.06–0.12)
QM 5	Maintained	Broad-spectrum antibiotics	DDDs of amoxicillin/clavulanate (J01CR02)/100 resident-days	1.35 (0.97–1.90)	1.20 (0.81–1.71)
QM 6	Maintained	Broad-spectrum antibiotics	Number of prescriptions of cephalosporins (J01D)/100 resident-days	0.07 (0.05–0.11)	0.11 (0.07–0.15)
QM 7	Maintained	Broad-spectrum antibiotics	DDDs of cephalosporins (J01D)/100 resident-days	0.30 (0.16–0.51)	0.48 (0.27–0.75)
QM 8	Maintained	Broad-spectrum antibiotics	Number prescriptions of quinolones (J01M)/100 resident-days	0.04 (0.02–0.05)	0.04 (0.03–0.06)
QM 9	Maintained	Broad-spectrum antibiotics	DDDs of quinolones (J01M)/100 resident-days	0.36 (0.20–0.55)	0.41 (0.25–0.61)
QM 10	Maintained	Second-line antibiotics	Number prescriptions of MLSK (J01F)/100 resident-days	0.05 (0.03–0.08)	0.05 (0.03–0.08)
QM 11	Maintained	Second-line antibiotics	DDDs of MLSK (J01F)/100 resident-days	0.54 (0.31–0.90)	0.52 (0.31–0.78)
QM 12	Maintained	Route of antibiotic administration	Number of prescriptions of parenteral antibiotic (J01 with IV, IM or SC route)/number of prescriptions of oral + parenteral antibiotics (J01)	8.33 (5.00–12.39)	10.75 (7.38–15.15)
QM 13	Maintained	Urine cultures prescriptions	Number of UC/100 resident-days	0.15 (0.07–0.22)	0.19 (0.12–0.28)
QM 14	Maintained	Urine cultures prescriptions	Number of residents (regardless their duration of stay) having at least 1 UC per year/total number of resident per year	17.91 (9.68–24.44)	23.86 (16.00–32.20)

IM, intramuscular; MLSK, macrolides, lincosamides, streptogramins and ketolides; SC, subcutaneous; UC, urine culture.

**Table 2.** Final set of 10 proxy indicators after consensus statement with Delphi method, and results calculated at the Nursing Home Level for two French areas

No.	Consensus	Field	Type	Calculation description	Target	Pays de la Loire area, median (IQR)	Lorraine area, median (IQR)
PI 1	Modified	Urinary tract infections	Ratio	In the week following UC, number of prescriptions of nitrofurantoin (J01XE01) + fosfomycin/trometamol (J01XX01) + pivmecillinam (J01CA08) + amoxicillin (J01CA04) + amoxicillin/clavulanate (J01CR02)/number of prescriptions of quinolones (J01M) + cephalosporins (J01D) + sulfamethoxazole/trimethoprim (J01EE01) for the year for male residents	Optimal 0 and acceptable <0.2	0.00 (0.00–10.00)	0.00 (0.00–8.33)
PI 2	Modified	Urinary tract infections	Ratio	In the week following UC, number of prescriptions of nitrofurantoin (J01XE01) + fosfomycin/trometamol (J01XX01) + pivmecillinam (J01CA08)/number of prescriptions of quinolones (J01M) for the year for female residents	>1.5	0.35 (0.00–0.86)	0.25 (0.00–0.59)
PI 3	Maintained	Repeated prescriptions of quinolones	%	Number of prescriptions of quinolones (J01M) among residents having been prescribed quinolones in the preceding 6 months/total number of prescriptions of quinolones for the year	Optimal 0 and acceptable <10%	14.29 (0.00–25.00)	19.52 (0.00–28.57)
PI 4	Maintained	Seasonal variation in antibiotic prescriptions	%	[Number of prescriptions of antibiotic (J01) during the cold-weather season (January–March and October–December)/number of prescriptions of antibiotic during the hot-weather season −1] × 100	<20%	28.44 (6.67–55.17)	36.90 (12.56–63.75)
PI 5	Modified	Seasonal variation in antibiotic prescriptions	%	[Number of prescriptions of amoxicillin/clavulanate (J01CR02) during the cold-weather season (January–March and October–December)/number of prescriptions of amoxicillin/clavulanate (J01CR02) during the hot-weather season −1] × 100	<20%	33.33 (0.00–100.00)	51.67 (0.00–140.00)
PI 6	Modified	First-line antibiotics versus second line antibiotics	Ratio	Number of prescriptions of amoxicillin (J01CA04) + amoxicillin/clavulanate (J01CR02)/number of prescriptions of quinolones (J01M) + cephalosporins (J01D) + MLSK (J01F)	>1.5	1.38 (1.04–1.78)	0.98 (0.73–1.39)
PI 7	Modified	Prolonged courses of antibiotics	Ratio	Number of prescriptions > 7 days for specific antibiotics^a^/total number of antibiotic prescriptions for these antibiotics	Optimal <5% and acceptable <20%	54.12 (43.55–61.54)	48.68 (40.38–56.62)
PI 8	Maintained	Co-prescriptions	%	Number of antibiotics (J01) + systemic NSAID (M01A) co-prescribed on the same day/total number of antibiotic prescriptions	Optimal 0 and acceptable <5%	0.00 (0.00–0.00)	0.00 (0.00–0.91)
PI 9	Modified	Flu vaccine coverage	%	Number of flu vaccines dispensed during the cold-weather season (January–March and October–December)/number of residents staying in the NH during the cold-weather season	≥90%	81.25 (74.36–88.14)	80.95 (70.73–88.24)
PI 10	Added	Route of antibiotic administration	%	Number of prescriptions of oral cephalosporins (J01D)/number of prescriptions of oral + parenteral (with IV, IM or SC route) cephalosporins (J01D)	Optimal <10% and acceptable <30%	60.00 (43.30–78.17)	57.14 (42.42–69.23)

IM, intramuscular; MLSK, macrolides, lincosamides, streptogramins and ketolides; NSAID, non-steroidal anti-inflammatory drug; SC, subcutaneous; UC, urine culture.

a Amoxicillin, amoxicillin/clavulanate, cefuroxime, cefpodoxime, roxithromycin, clarithromycin, pristinamycin, nitrofurantoin.

### Practical use of selected indicators for the national and local monitoring of antibiotic prescriptions

The indicators were assessed using the NHI databases from two French administrative areas: the Grand Est and the Pays de la Loire regions. In 2019, there were 417 NHs in the Grand Est and 443 in the Pays de la Loire regions. The mean number (±SD) of residents per NH was 91.5 ± 36.2 in Grand Est and 93.8 ± 32.2 in Pays de la Loire. The mean age of residents was, respectively, 87.3 ± 7.3 and 88.3 ± 1.8 years, and 75.2% and 74.6% were women. QM and PI results (median and IQR), for the year 2019 in both areas, are presented in Tables 1 and 2. Indicators were consistent across both areas. Wide variations were noted between NHs for all QMs.

**Figure 2.**
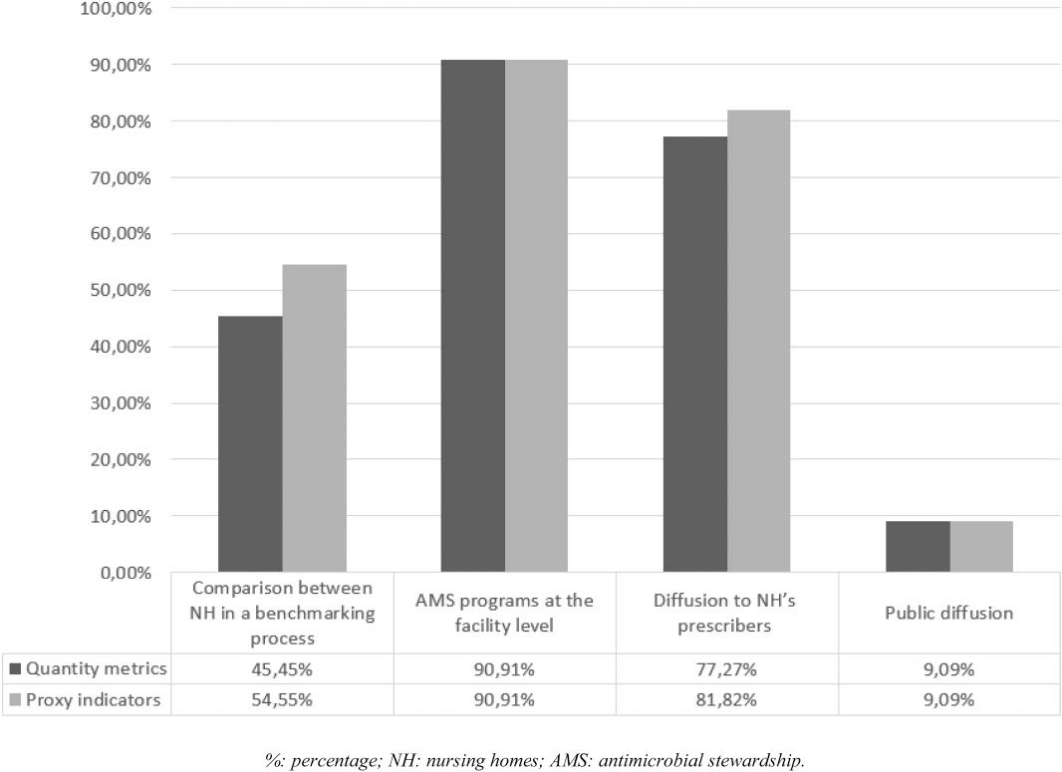
Possible uses for QMs and PIs.

On an exploratory basis, the 20 experts were asked to give their opinion regarding possible uses of the indicators, and 11 completed the survey. Most of the experts who answered (91%) were in favour of sharing the indicators within the French regional AMS network for local programmes at the facility level (Figure 2). The second and third uses of indicators were feedback to NH prescribers (82%) and to benchmark NHs through regional and national health authorities (55%). Public reporting was poorly rated, with only 9% of experts in favour of this use of the indicators.

## Discussion

### Development of a relevant set of indicators for AMS in NHs

This study provides a consensual list of 14 QMs and 10 PIs adapted to AMS in NHs, for estimating the volume and appropriateness of antimicrobial prescriptions, respectively, in NHs based on national reimbursement data.

This list of indicators includes a wide range of frequent clinical situations in NHs, from overuse to misuse of antibiotics. The overuse of antibiotics was approached by 14 indicators, 11 QMs and 3 PIs. Monitoring the prescription of urine cultures is a way of addressing the treatment of asymptomatic bacteriuria. Regarding upper respiratory tract infections in primary care, 80% of treatment courses may exceed the recommended duration.^24^ To overcome the unavailability of prescription durations in the NHI database, an estimation was performed using the quantity of packages dispensed. In the panel discussion, the target initially set at >8 days (to offset the excess of pills dispensed) was lowered to >7 days. This choice was made to be in accordance with the guidelines, provide a more understandable message for AMS programmes, and consider the recent actualization of French guidelines for antibiotic duration,^23^ which reduced treatment courses to 5 days in some upper respiratory tract infections. Increased antibiotic prescription during winter is a reality, especially for respiratory tract infections,^25^ which are more frequent in winter but mostly viral.^26,27^

One QM and five PIs focused on the misuse of antibiotics. Beyond the monitoring of broad-spectrum antibiotics, it is essential that the duration, route of administration and recurrence of prescriptions be addressed. The use of oral cephalosporins that may generate suboptimal concentrations,^28^ repeated prescriptions of quinolones within a 6 month period^29^ and the choice of drugs for first-line regimens have all been associated with an increased risk of AMR. An indicator related to flu vaccination falls outside the scope of antibiotic reimbursement, but is also available in SNDS database, and is indirectly correlated with antibiotic prescriptions, since flu vaccination might prevent one in 25 antibiotic prescriptions among acute respiratory infections during the influenza season.^30^ Monitoring and benchmarking these practices may lead to improved use of antibiotics during winter.

### Practical use of selected indicators for the national and local monitoring of antibiotic prescriptions

This list of indicators developed and validated for French NHs is easy to generate from NHI databases and may be of use at the international scale for benchmarking purposes. These outputs will contribute to the national monitoring of antibiotic use in French NHs, in line with the 2022–25 national strategy for preventing antibiotic resistance.^31^ The experts were asked to give their opinion about possible uses for the indicators. Unfortunately, only 11 of the 20 experts completed the survey, so the results have to be taken with caution. Regarding PIs, 91% of our experts supported transmitting them to regional AMS networks for local programmes at the facility level. Several stakeholders, such as the Regional and National Health Insurance organization and the regional antibiotic stewardship networks, might be able to use these data to help NHs develop personalized action plans, with concrete objectives and a monitoring plan. Moreover, 82% were inclined to transmit them to NH prescribers. Almost real-time personalized feedback for prescribers, or integration into a pay-for-performance mechanism, which exists in France for GPs at the individual level but not collectively at the NH level, are strategies worth considering in coming years. Only 55% of experts voted for transmission to Regional Health Authorities for benchmarking purposes. One reason lies in France’s hierarchical health governance, with Regional Health Authorities appearing to be organizations responsible more for regulations, inspections and controls than advice.^32^ Despite common transparent approaches open to public scrutiny in France, only 9% of experts supported public reporting of the indicators. Public reporting has been associated with increased quality-improvement activity.^33^ However, this strategy is exposed to the risk of the indicators reported being misunderstood by the public or media.

### Conclusions

In this study, 14 QMs and 10 PIs based on the reimbursement database were selected and validated to monitor the use of antibiotics in French NHs. This consensual list of indicators may improve practices at the local, regional and national scale.

## Supplementary Material

dlad037_Supplementary_Data

## Data Availability

The original and anonymous dataset is available from the corresponding author upon request.

## Members of the French expert group for AMS in NHs

Joël Ankri, Philippe Benoît, Sabine Benoliel, Anne Berger-Carbonne, Willy Boutfol, Philippe Cavalie, Bernard Clary, Hélène Cormier, Matthieu Coulongeat, Benoit De Wazieres, Sylvain Diamantis, Catherine Dumartin, Gaël Durel, Chantal Erault, Thibault Fraisse, Agnès Gaudichon, Françoise Jay-Rayon, Amélie Jouzeau, Garmenick Leblanc, Anne-Sophie Lelong, Joël Leroy, Frédéric Maraval, Nathalie Maubourguet, Béatrice Van Oost, Patricia Pavese, Emmanuel Piednoir, Odile Reynaud-Levy, Béatrice Rosolen and Adeline Welter.

## Supplementary data

Data files S1 and S2 and Tables S1 to S3 are available as Supplementary data at *JAC-AMR* Online.
