# Supplementary material for: Expert consensus on monitoring antimicrobial stewardship in French nursing homes using assessed reimbursement database indicators
Source: JAC Antimicrob Resist. 2023 Mar 31;5(2):dlad037. doi: 10.1093/jacamr/dlad037 (PMC10064325; doi:10.1093/jacamr/dlad037)
Supplement: dlad037_Supplementary_Data [file dlad037_supplementary_data.zip › Supplementary Tables.docx]

**Supplementary Table 1: list of experts and observers composing the national committee**

| Experts |
| --- |
| Collège de médecine générale (CMG) Dr Bernard Clary |
| Société Française de gériatrie et gérontologie (SFGG) Pr Benoit De Wazieres et Dr Matthieu Coulongeat |
| Fédération française des associations de médecins coordonnateurs d’EHPAD (FFAMCO) Dr Nathalie Maubourguet |
| Association nationale des médecins coordonnateurs et du secteur médico-social (MCOOR) Dr Gaël Durel, Dr Frédéric Maraval et Dr Odile Reynaud-Levy |
| Société de pathologie infectieuse de langue française (SPILF)  Dr Sylvain Diamantis, Dr Thibault Fraisse et Pr David Boutoille |
| Centres régionaux en antibiothérapie (CRAtb) Dr Willy Boutfol, Dr Hélène Cormier (Pays de la Loire), Dr Joël Leroy, Dr Béatrice Rosolen (Bourgogne-Franche-Comté), Dr Patricia Pavese (Auvergne-Rhône-Alpes). |
| Réseau de prévention des infections associées aux soins (RéPIAS) Dr Agnès Gaudichon (Ile-de-France) et Dr Emmanuel Piednoir (Bretagne) |
| Conseil national de l’ordre des pharmaciens (CNOP) Dr Philippe Benoît |
| Mission Spares de Santé Publique France (SPF) Dr Catherine Dumartin et Dr Amélie Jouzeau |
| Observers |
| Caisse nationale d’assurance maladie (CNAM) Pr Joël Ankri, Dr Béatrice Van Oost, Mme Garmenick Leblanc et Mme Anne-Sophie Lelong |
| Assurance maladie (DRSM)  Dr Anicet Chaslerie (Pays de la Loire), Mme Ouarda Pereira et Mme Adeline Welter (Grand Est) |
| Ministère des solidarités et de la santé (MSS) Pr Céline Pulcini |
| Direction générale de la cohésion sociale (DGCS) Dr Chantal Erault et Françoise Jay-Rayon |
| Haute autorité de santé (HAS) Dr Sabine Benoliel |
| Santé publique France (SPF)  Dr Anne Berger-Carbonne et Dr Philippe Cavalie |
| Analysts |
| Mission Primo de Santé Publique France (SPF) Dr Antoine Asquier-Khati, Dr Colin Deschanvres et Dr Gabriel Birgand |

**Supplementary Table 2: evaluation of quantity metrics by Likert’s scale during the first round**

| **Calcul description** | | **Maximal note (excellent)** | **Mean** | **Global evaluation** |
| --- | --- | --- | --- | --- |
| QM 1 | Number of antibiotic prescriptions / 100 resident-days | N = 5 | 4.08 | ✱✱✱ |
| QM 2 | DDDs of antibiotics / 100 residents-days | N = 3 | 3.50 | ✱ |
| QM 3 | Number of residents receiving at least 1 antibiotic per year / total number of residents per year | N = 5 | 4.08 | ✱✱✱ |
| QM 4 | Number of antibiotic prescriptions / total number of residents per year | N = 4 | 3.83 | ✱✱ |
| QM 5 | Number of prescriptions of amoxicillin-clavulanate + quinolones + cephalosporins / 100 resident-days | N = 3 | 3.5 | ✱ |
| QM 6 | DDDs of amoxicillin-clavulanate + quinolones + cephalosporins / 100 resident-days | N = 4 | 3.91 | ✱✱ |
| QM 7 | Number of prescriptions of amoxicillin-clavulanate / 100 resident-days | N = 6 | 4.16 | ✱✱✱ |
| QM 8 | DDDs of amoxicillin-clavulanate / 100 resident-days | N = 4 | 3.67 | ✱✱ |
| QM 9 | Number of prescriptions of cephalosporins / 100 resident-days | N = 5 | 3.83 | ✱✱ |
| QM 10 | DDDs of cephalosporins / 100 resident-days | N = 2 | 3.25 | ✱ |
| QM 11 | Number prescriptions of quinolones / 100 resident-days | N = 5 | 4.09 | ✱✱✱ |
| QM 12 | DDDs of quinolones / 100 resident-days | N = 3 | 3.73 | ✱✱ |
| QM 13 | Number prescriptions of MLSK / 100 resident-days | N = 4 | 4.00 | ✱✱ |
| QM 14 | DDDs of MLSK / 100 resident-days | N = 2 | 3.30 | ✱ |
| QM 15 | Number of prescriptions of topical antibiotics / 100 resident-days | N = 6 | 4.18 | ✱✱✱ |
| QM 16 | Number of prescriptions of parenteral antibiotic (with IV, IM or SC route) / number of prescriptions of oral + parenteral antibiotics | N = 5 | 3.67 | ✱✱ |
| QM 17 | Number of prescriptions of more than 1 antibiotic on the same day / number of antibiotic prescriptions | N = 3 | 3.67 | ✱✱ |
| QM 18 | Number of prescriptions of antibiotics with a different antibiotic prescribed the week after the first prescription / number of antibiotic prescription | N = 3 | 3.36 | ✱ |
| QM 19 | Number of urine cultures / 100 resident-days | N = 6 | 4.08 | ✱✱✱ |
| QM 20 | Number of residents (regardless their duration of stay) having at least 1 urine culture per year / total number of resident per year | N = 8 | 4.27 | ✱✱✱ |
| QM 21 | Number of urine cultures / total number of residents per year | N = 4 | 3.45 | ✱✱ |

*DDD: defined daily doses; MLSK: macrolides, lincosamides, streptogramins and ketolides; Maximal note: number of times the indicator achieves the best note (Excellent); Mean: conversion of Likert scale from 1 to 5; Global evaluation: ✱ fair indicator (mean ≤ 3.5), ✱✱ good indicator (mean > 3.5 et ≤ 4), ✱✱✱ excellent indicator (mean > 4).*

**Supplementary Table 3: evaluation of proxy indicators by Likert’s scale during the first round**

| **Calcul description** | | **Maximal note (excellent)** | **Mean** | **Global evaluation** |
| --- | --- | --- | --- | --- |
| PI 1 | Number of prescriptions of nitrofurantoin + certain quinolones^a^ + fosfomycin-trometamol / number of prescriptions of antibiotics for the year for male resident | N = 2 | 3.91 | ✱✱ |
| PI 2 | Number of prescriptions of nitrodurantoin + pivmecillinam + fosfomycine-trometamol / number of prescriptions of quinolones for the year for female residents | N = 4 | 3.73 | ✱✱ |
| PI 3 | Number of prescriptions of quinolones among residents having been prescribed quinolones in the preceding 6 months / total number of prescriptions of quinolones for the year | N = 7 | 4.55 | ✱✱✱ |
| PI 4 | [Number of prescriptions of antibiotic during the cold-weather season (january-march and october-december) / number of prescriptions of antibiotic during the hot-weather season -1] x 100 | N = 4 | 3.67 | ✱✱ |
| PI 5 | [Number of prescriptions of quinolones during the cold-weather season (january-march and october-december) / number of prescriptions of quinolones during the hot-weather season -1] x 100 | N = 0 | 2.83 | ✱ |
| PI 6 | Number of prescriptions of amoxicillin + amoxicillin-clavulanate / number of prescriptions of quinolones + cephalosporins + MLSK | N = 4 | 3.67 | ✱✱ |
| PI 7 | Number of prescriptions of lomefloxacin + moxifloxacin + certains quinolones* + telithromycin + spiramycin-metronidazole + cefaclor + cefadroxil / total number of antibiotic prescriptions | N = 3 | 3.72 | ✱✱ |
| PI 8 | Number of prescriptions > 8 days for specific antibiotics ^b^  / total number of antibiotic prescriptions for these antibiotics | N = 5 | 4.25 | ✱✱✱ |
| PI 9 | Number of antibiotics + systemic NSAID co-prescribed on the same day / total number of antibiotic prescriptions | N = 6 | 4.27 | ✱✱✱ |
| PI 10 | Number of antibiotics + systemic corticosteroids co-prescribed on the same day / total number of antibiotic prescriptions | N = 4 | 4.09 | ✱✱✱ |
| PI 11 | Number of flu vaccines dispensed during the second semester / number of residents staying in the NH between october and december | N = 7 | 4.83 | ✱✱✱ |

*MLSK: macrolides, lincosamides, streptogramins and ketolides; NH: nursing homes; NSAID: nonsteroidal anti-inflammatory drug; Maximal note: number of times the indicator achieves the best note (Excellent); Mean: conversion of Likert scale from 1 to 5; Global evaluation: ✱ fair indicator (mean ≤ 3.5), ✱✱ good indicator (mean > 3.5 et ≤ 4), ✱✱✱ excellent indicator (mean > 4).*
