# Supplementary material for: Expert consensus on monitoring antimicrobial stewardship in French nursing homes using assessed reimbursement database indicators
Source: JAC Antimicrob Resist. 2023 Mar 31;5(2):dlad037. doi: 10.1093/jacamr/dlad037 (PMC10064325; doi:10.1093/jacamr/dlad037)
Supplement: dlad037_Supplementary_Data [file dlad037_supplementary_data.zip › Supplementary Data 1.docx]

**Supplementary data 1**

**Web-based questionnaire to evaluate Quantity Metrics and Proxy Indicators
in Nursing Homes**

**QUANTITY METRICS :**For each indicator, rate its relevance for assessing antibiotic consumption in Nursing Homes

1. **Number of antibiotics prescriptions / 100 resident-days.**

Score from 1 (not at all relevant) to 5 (very relevant) : 1 ; 2 ; 3 ; 4 ; 5 ;

If relevance rated between 1 and 3, explain why. If relevance rated between 4 and 5, does the definition of the indicator (numerator, denominator, target population) suit you? Would you make suggestions for improvement (specify)?

………………………………………………………………………………………………………………………………………………………..

1. **DDDs of antibiotics / 100 resident-days.**

Score from 1 (not at all relevant) to 5 (very relevant) : 1 ; 2 ; 3 ; 4 ; 5 ;

If relevance rated between 1 and 3, explain why. If relevance rated between 4 and 5, does the definition of the indicator (numerator, denominator, target population) suit you? Would you make suggestions for improvement (specify)?

………………………………………………………………………………………………………………………………………………………..

1. **Number of residents receiving at least 1 antibiotic per year / total number of resident per year.**

Score from 1 (not at all relevant) to 5 (very relevant) : 1 ; 2 ; 3 ; 4 ; 5 ;

If relevance rated between 1 and 3, explain why. If relevance rated between 4 and 5, does the definition of the indicator (numerator, denominator, target population) suit you? Would you make suggestions for improvement (specify)?

………………………………………………………………………………………………………………………………………………………..

1. **Number of antibiotics prescriptions / total number of residents per year.**

Score from 1 (not at all relevant) to 5 (very relevant) : 1 ; 2 ; 3 ; 4 ; 5 ;

If relevance rated between 1 and 3, explain why. If relevance rated between 4 and 5, does the definition of the indicator (numerator, denominator, target population) suit you? Would you make suggestions for improvement (specify)?

………………………………………………………………………………………………………………………………………………………..

1. **Number of prescriptions of amoxicillin-clavulanate + quinolones + cephalosporins / 100 resident-days.**

Score from 1 (not at all relevant) to 5 (very relevant) : 1 ; 2 ; 3 ; 4 ; 5 ;

If relevance rated between 1 and 3, explain why. If relevance rated between 4 and 5, does the definition of the indicator (numerator, denominator, target population) suit you? Would you make suggestions for improvement (specify)?

………………………………………………………………………………………………………………………………………………………..

1. **DDDs of amoxicillin-clavulanate + quinolones + cephalosporins / 100 resident-days.**

Score from 1 (not at all relevant) to 5 (very relevant) : 1 ; 2 ; 3 ; 4 ; 5 ;

If relevance rated between 1 and 3, explain why. If relevance rated between 4 and 5, does the definition of the indicator (numerator, denominator, target population) suit you? Would you make suggestions for improvement (specify)?

………………………………………………………………………………………………………………………………………………………..

1. **Number of prescriptions of amoxicillin-clavulanate / 100 resident-days.**

Score from 1 (not at all relevant) to 5 (very relevant) : 1 ; 2 ; 3 ; 4 ; 5 ;

If relevance rated between 1 and 3, explain why. If relevance rated between 4 and 5, does the definition of the indicator (numerator, denominator, target population) suit you? Would you make suggestions for improvement (specify)?

………………………………………………………………………………………………………………………………………………………..

1. **DDDs of amoxicillin-clavulanate / 100 resident-days.**

Score from 1 (not at all relevant) to 5 (very relevant) : 1 ; 2 ; 3 ; 4 ; 5 ;

If relevance rated between 1 and 3, explain why. If relevance rated between 4 and 5, does the definition of the indicator (numerator, denominator, target population) suit you? Would you make suggestions for improvement (specify)?

………………………………………………………………………………………………………………………………………………………..

1. **Number of prescriptions of cephalosporins / 100 resident-days.**

Score from 1 (not at all relevant) to 5 (very relevant) : 1 ; 2 ; 3 ; 4 ; 5 ;

If relevance rated between 1 and 3, explain why. If relevance rated between 4 and 5, does the definition of the indicator (numerator, denominator, target population) suit you? Would you make suggestions for improvement (specify)?

………………………………………………………………………………………………………………………………………………………..

1. **DDDs of cephalosporins / 100 resident-days.**

Score from 1 (not at all relevant) to 5 (very relevant) : 1 ; 2 ; 3 ; 4 ; 5 ;

If relevance rated between 1 and 3, explain why. If relevance rated between 4 and 5, does the definition of the indicator (numerator, denominator, target population) suit you? Would you make suggestions for improvement (specify)?

………………………………………………………………………………………………………………………………………………………..

1. **Number of prescriptions of quinolones / 100 resident-days.**

Score from 1 (not at all relevant) to 5 (very relevant) : 1 ; 2 ; 3 ; 4 ; 5 ;

If relevance rated between 1 and 3, explain why. If relevance rated between 4 and 5, does the definition of the indicator (numerator, denominator, target population) suit you? Would you make suggestions for improvement (specify)?

………………………………………………………………………………………………………………………………………………………..

1. **DDDs of quinolones / 100 resident-days.**

Score from 1 (not at all relevant) to 5 (very relevant) : 1 ; 2 ; 3 ; 4 ; 5 ;

If relevance rated between 1 and 3, explain why. If relevance rated between 4 and 5, does the definition of the indicator (numerator, denominator, target population) suit you? Would you make suggestions for improvement (specify)?

………………………………………………………………………………………………………………………………………………………..

1. **Number of prescriptions of MLSK / 100 resident-days.**

Score from 1 (not at all relevant) to 5 (very relevant) : 1 ; 2 ; 3 ; 4 ; 5 ;

If relevance rated between 1 and 3, explain why. If relevance rated between 4 and 5, does the definition of the indicator (numerator, denominator, target population) suit you? Would you make suggestions for improvement (specify)?

………………………………………………………………………………………………………………………………………………………..

1. **DDDs of MLSK / 100 resident-days.**

Score from 1 (not at all relevant) to 5 (very relevant) : 1 ; 2 ; 3 ; 4 ; 5 ;

If relevance rated between 1 and 3, explain why. If relevance rated between 4 and 5, does the definition of the indicator (numerator, denominator, target population) suit you? Would you make suggestions for improvement (specify)?

………………………………………………………………………………………………………………………………………………………..

1. **Number of prescriptions of topical antibiotics / 100 resident-days.**

Score from 1 (not at all relevant) to 5 (very relevant) : 1 ; 2 ; 3 ; 4 ; 5 ;

If relevance rated between 1 and 3, explain why. If relevance rated between 4 and 5, does the definition of the indicator (numerator, denominator, target population) suit you? Would you make suggestions for improvement (specify)?

………………………………………………………………………………………………………………………………………………………..

1. **Number of prescriptions of parenteral antibiotic / number of prescriptions of oral + parenteral antibiotics.**

Score from 1 (not at all relevant) to 5 (very relevant) : 1 ; 2 ; 3 ; 4 ; 5 ;

If relevance rated between 1 and 3, explain why. If relevance rated between 4 and 5, does the definition of the indicator (numerator, denominator, target population) suit you? Would you make suggestions for improvement (specify)?

………………………………………………………………………………………………………………………………………………………..

1. **Number of prescriptions of more than 1 antibiotic on the same day / number of antibiotic prescriptions**

Score from 1 (not at all relevant) to 5 (very relevant) : 1 ; 2 ; 3 ; 4 ; 5 ;

If relevance rated between 1 and 3, explain why. If relevance rated between 4 and 5, does the definition of the indicator (numerator, denominator, target population) suit you? Would you make suggestions for improvement (specify)?

………………………………………………………………………………………………………………………………………………………..

1. **Number of prescriptions of antibiotics with a different antibiotic prescribed the week after the first prescription / number of antibiotic prescriptions**

Score from 1 (not at all relevant) to 5 (very relevant) : 1 ; 2 ; 3 ; 4 ; 5 ;

If relevance rated between 1 and 3, explain why. If relevance rated between 4 and 5, does the definition of the indicator (numerator, denominator, target population) suit you? Would you make suggestions for improvement (specify)?

………………………………………………………………………………………………………………………………………………………..

1. **Number of urine cultures / 100 resident-days.**

Score from 1 (not at all relevant) to 5 (very relevant) : 1 ; 2 ; 3 ; 4 ; 5 ;

If relevance rated between 1 and 3, explain why. If relevance rated between 4 and 5, does the definition of the indicator (numerator, denominator, target population) suit you? Would you make suggestions for improvement (specify)?

………………………………………………………………………………………………………………………………………………………..

1. **Number of residents having at least 1 urine culture per year / total number of residents per year.**

Score from 1 (not at all relevant) to 5 (very relevant) : 1 ; 2 ; 3 ; 4 ; 5 ;

If relevance rated between 1 and 3, explain why. If relevance rated between 4 and 5, does the definition of the indicator (numerator, denominator, target population) suit you? Would you make suggestions for improvement (specify)?

………………………………………………………………………………………………………………………………………………………..

1. **Number of urine cultures / total number of residents per year.**

Score from 1 (not at all relevant) to 5 (very relevant) : 1 ; 2 ; 3 ; 4 ; 5 ;

If relevance rated between 1 and 3, explain why. If relevance rated between 4 and 5, does the definition of the indicator (numerator, denominator, target population) suit you? Would you make suggestions for improvement (specify)?

………………………………………………………………………………………………………………………………………………………..

**PROXY INDICATORS :**For each indicator, rate its interest in evaluating the relevance of antibiotic prescriptions at nursing home level. We suggest that participants read the scientific evidence based supporting the definition of each proxy indicators detailed in attachments No.3 and No.4.

1. **Antibiotic prescriptions against UTI in men: number of prescriptions of nitrofurantoin + certain quinolones (norfloxacin, enoxacin, lomefloxacin) + fosfomycin-trometamol / number of prescriptions of antibiotics for the year for male residents. Optimal target 0 and acceptable target < 0.5.**

Score from 1 (not at all relevant) to 5 (very relevant) : 1 ; 2 ; 3 ; 4 ; 5 ;

If relevance rated between 1 and 3, explain why. If relevance rated between 4 and 5, does the definition of the indicator (numerator, denominator, target population) suit you? Would you make suggestions for improvement (specify)? Does the proposed target value suit you? Or would you like to suggest another target (specify)?

………………………………………………………………………………………………………………………………………………………..

1. **Antibiotic prescriptions against UTI in women: number of prescriptions of nitrofurantoin + pivmecillinam + fosfomycin-trometamol / number of prescriptions of quinolones for the year for female residents. Target > 1.**

Score from 1 (not at all relevant) to 5 (very relevant) : 1 ; 2 ; 3 ; 4 ; 5 ;

If relevance rated between 1 and 3, explain why. If relevance rated between 4 and 5, does the definition of the indicator (numerator, denominator, target population) suit you? Would you make suggestions for improvement (specify)? Does the proposed target value suit you? Or would you like to suggest another target (specify)?

………………………………………………………………………………………………………………………………………………………..

1. **Repeated prescription of quinolones: number of prescriptions of quinolones among residents having been prescribed quinolones in the preceding 6 months / total number of prescriptions of quinolones. Optimal target 0 and acceptable target < 10%.**

Score from 1 (not at all relevant) to 5 (very relevant) : 1 ; 2 ; 3 ; 4 ; 5 ;

If relevance rated between 1 and 3, explain why. If relevance rated between 4 and 5, does the definition of the indicator (numerator, denominator, target population) suit you? Would you make suggestions for improvement (specify)? Does the proposed target value suit you? Or would you like to suggest another target (specify)?

………………………………………………………………………………………………………………………………………………………..

1. **Seasonal variation in total antibiotic prescriptions: [number of prescriptions of antibiotic during the cold weather season (january-march and october-december) / number of prescriptions of antibiotic during the hot weather season (april-september) - 1] x 100. Target < 20%.**

Score from 1 (not at all relevant) to 5 (very relevant) : 1 ; 2 ; 3 ; 4 ; 5 ;

If relevance rated between 1 and 3, explain why. If relevance rated between 4 and 5, does the definition of the indicator (numerator, denominator, target population) suit you? Would you make suggestions for improvement (specify)? Does the proposed target value suit you? Or would you like to suggest another target (specify)?

………………………………………………………………………………………………………………………………………………………..

1. **Seasonal variation in quinolones prescriptions: [number of prescriptions of quinolones during the cold weather season (january-march and october-december) / number of prescriptions of quinolones during the hot weather season (april-september) - 1] x 100. Optimal target < 5% and acceptable target < 10%.**

Score from 1 (not at all relevant) to 5 (very relevant) : 1 ; 2 ; 3 ; 4 ; 5 ;

If relevance rated between 1 and 3, explain why. If relevance rated between 4 and 5, does the definition of the indicator (numerator, denominator, target population) suit you? Would you make suggestions for improvement (specify)? Does the proposed target value suit you? Or would you like to suggest another target (specify)?

………………………………………………………………………………………………………………………………………………………..

1. **First-line antibiotics / second-line antibiotics prescriptions: number of prescriptions of amoxicillin-clavulanate / number of prescriptions of quinolones + cephalosporins + MLSK. Target > 1/**

Score from 1 (not at all relevant) to 5 (very relevant) : 1 ; 2 ; 3 ; 4 ; 5 ;

If relevance rated between 1 and 3, explain why. If relevance rated between 4 and 5, does the definition of the indicator (numerator, denominator, target population) suit you? Would you make suggestions for improvement (specify)? Does the proposed target value suit you? Or would you like to suggest another target (specify)?

………………………………………………………………………………………………………………………………………………………..

1. **Prescriptions of not indicated antibiotics: number of prescriptions of lomefloxacin + moxifloxacin + certain quinolones (norfloxacin, enoxacin, lomefloxacin) + telithromycin + spiramycin-metronidazole + cefaclor + cefadroxil / total number of antibiotic prescriptions. Optimal target 0 and acceptable target < 0.5.**

Score from 1 (not at all relevant) to 5 (very relevant) : 1 ; 2 ; 3 ; 4 ; 5 ;

If relevance rated between 1 and 3, explain why. If relevance rated between 4 and 5, does the definition of the indicator (numerator, denominator, target population) suit you? Would you make suggestions for improvement (specify)? Does the proposed target value suit you? Or would you like to suggest another target (specify)?

………………………………………………………………………………………………………………………………………………………..

1. **Estimated duration of antibiotic prescriptions > 8 days: Number of prescriptions > 8 days for specific antibiotics (see attachment No.4) / total number of antibiotic prescriptions for these antibiotics. Optimal target < 5% and acceptable target < 10%.**

Score from 1 (not at all relevant) to 5 (very relevant) : 1 ; 2 ; 3 ; 4 ; 5 ;

If relevance rated between 1 and 3, explain why. If relevance rated between 4 and 5, does the definition of the indicator (numerator, denominator, target population) suit you? Would you make suggestions for improvement (specify)? Does the proposed target value suit you? Or would you like to suggest another target (specify)?

………………………………………………………………………………………………………………………………………………………..

1. **Co-prescription of antibiotic + systemic NSAIDs: number of antibiotic + systemic NSAIDs co-prescribed on the same day / total number of antibiotic prescriptions. Optimal target 0 and acceptable target < 5%.**

Score from 1 (not at all relevant) to 5 (very relevant) : 1 ; 2 ; 3 ; 4 ; 5 ;

If relevance rated between 1 and 3, explain why. If relevance rated between 4 and 5, does the definition of the indicator (numerator, denominator, target population) suit you? Would you make suggestions for improvement (specify)? Does the proposed target value suit you? Or would you like to suggest another target (specify)?

………………………………………………………………………………………………………………………………………………………..

1. **Co-prescription of antibiotic + systemic corticosteroids: number of antibiotic + systemic corticosteroids co-prescribed on the same day / total number of antibiotic prescriptions. Optimal target 0 and acceptable target < 5%.**

Score from 1 (not at all relevant) to 5 (very relevant) : 1 ; 2 ; 3 ; 4 ; 5 ;

If relevance rated between 1 and 3, explain why. If relevance rated between 4 and 5, does the definition of the indicator (numerator, denominator, target population) suit you? Would you make suggestions for improvement (specify)? Does the proposed target value suit you? Or would you like to suggest another target (specify)?

………………………………………………………………………………………………………………………………………………………..

1. **Estimated flu vaccine coverage: number of flu vaccines dispensed during the second semester / number of residents staying in the NH between October and December. Target ≥ 90%.**

Score from 1 (not at all relevant) to 5 (very relevant) : 1 ; 2 ; 3 ; 4 ; 5 ;

If relevance rated between 1 and 3, explain why. If relevance rated between 4 and 5, does the definition of the indicator (numerator, denominator, target population) suit you? Would you make suggestions for improvement (specify)? Does the proposed target value suit you? Or would you like to suggest another target (specify)?

………………………………………………………………………………………………………………………………………………………..
