# Supplementary material for: Expert consensus on monitoring antimicrobial stewardship in French nursing homes using assessed reimbursement database indicators
Source: JAC Antimicrob Resist. 2023 Mar 31;5(2):dlad037. doi: 10.1093/jacamr/dlad037 (PMC10064325; doi:10.1093/jacamr/dlad037)
Supplement: dlad037_Supplementary_Data [file dlad037_supplementary_data.zip › Supplementary Data 2.docx]

**Supplementary Data 2**

**National meeting for the validation of Quantity Indicators and Proxy Indicators
in nursing homes**

**Objective of the meeting:**

Validate by a national working group the quantity indicators and proxy indicators to be used in nursing homes for monitoring the consumption and the appropriateness of antibiotic prescriptions in France.

**Rationale of the project:**

In France, the appropriate use of antibiotics (1), mainly studied in hospitals, has been the subject of only a few studies in medico-social establishments, particularly in nursing homes (2). Nursing homes residents nevertheless represent a large and ever-increasing part of the general French population, frequently exposed to antibiotics. The overconsumption and misuse of antibiotics are likely to increase antibiotic selection pressure and promote the acquisition of resistant strains (3). In nursing homes, the consumption of antibiotics, in median Defined Daily Dose (DDD), is estimated at more than 40 per 1000 residents/day (4), with a percentage of misuse estimated at more than 50% for some studies (5). At the same time, antibiotic resistance is higher in nursing homes: for example, the Primo network evaluated in 2019 via MedQual-Ville, the percentage of ESBL *E. coli* in urine cultures at 8.7% for Nursing homes residents compared to 3.0% in primary care (6). Actions against antibiotic resistance in Nursing homes, based on correct prescriptions of antibiotics, require a continuous and standardized evaluation of the consumption of antibiotics in Nursing homes, in order to set medium and long-term objectives to improve practices.

Recently, a study (7) in Lorraine (Attachment No.1) analysed the consumption of antibiotics in 209 nursing homes on the basis of health insurance reimbursement data, using two types of indicators: quantity indicators (example : number of antibiotic prescriptions / 100 resident-days) and indirect indicators, called proxy indicators (example: number of re-prescriptions of quinolones in a patient who has already received quinolones in the last 6 months), more likely to estimate the appropriateness of antibiotic prescriptions at the Nursing Home level. These proxy-indicators are the result of a systematic review of the literature and an expert consensus (8). Eleven proxy-indicators have been developed (see list of proxy-indicators) with, for each, target values ​​to be achieved with the objective of appropriate use of antibiotics based on national recommendations. The proxy-indicators were then evaluated according to three clinimetric properties: measurability (data available for more than 75% of EHPADs), applicability (proxy indicator calculable at least 10 times for at least 75% of EHPADs), and room for improvement (considered insufficient if < 15%). In the end, six proxy indicators were selected for their good clinimetric properties. Proxy indicators 2-3-5-7 were excluded because applicable for less than 75% of EHPADs? Proxy indicator 9 was also not retained due to a margin for improvement of less than 15%. Data were available and measurable in 100% of cases. Finally, nursing homes could be classified into 3 groups according to their ability to reach the target values ​​for the 6 proxy indicators selected: target value reached for ≤ 1 proxy indicator (1st group), for 2 proxy indicators (2nd group), and for ≥ 3 proxy indicators (3rd group).

The objective is now to apply these quantitative indicators and these proxy indicators for all Nursing homes in France. With this in mind, we propose to compose a national working group representing several organizations and learned societies implicated in antibiotic prescriptions (CMG, SFGG, FFAMCO, MCOOR, SPILF, CNAM, HAS, MSS, SpF), in order to allow discuss and then validate of a definitive list of indicators that will be retained at the national level. The use of these indicators would allow the Regional Antibiotic Therapy Centers to propose, for each nursing homes, a feedback on their uses of antibiotic therapy, in order to define regional objectives and put in place an action plan to improve the appropriate use of antibiotics, all in conjunction with local players (nursing homes coordinating doctors, mobile antibiotic therapy teams, etc.).

**Questions posed to the national working group:**

We suggest that each participant read the document and send us feedback in the following two ways:

First by comments and suggestions directly on the martyr document. Secondly by evaluating each indicator (Likert scale from 1 to 5) on the Google Forms provided for this purpose: https://forms.gle/qBVbwG81Kuz9gMvm8. You will be asked to justify the answer provided, by detailing the parameters deemed relevant or open to criticism (numerator, denominator, target population, etc.). It will also be asked to evaluate and propose improvement suggestions for the target values ​​assigned to each proxy indicator.

We would like comments on the martyr document and answers to the Google Forms questionnaire to reach us no later than February 1, 2021.

**Procedure:**

• **January 5, 2021:**
Sending of the document and the questionnaire to the national group.

• **February 1, 2021:**
Deadline for completing the Google Forms questionnaire and sending us feedback and comments on the document.

• **February 22, 2021 from 3 p.m. to 5 p.m.:**
National meeting for the discussion of indicators by videoconference.

**List of Quantity Indicators to assess antibiotic consumption in Nursing homes:**

1. **Number of antibiotics prescriptions / 100 resident-days.**
2. **DDDs of antibiotics / 100 resident-days.**
3. **Number of residents receiving at least 1 antibiotic per year / total number of resident per year.**
4. **Number of antibiotics prescriptions / total number of residents per year.**
5. **Number of prescriptions of amoxicillin-clavulanate + quinolones + cephalosporins / 100 resident-days.**
6. **DDDs of amoxicillin-clavulanate + quinolones + cephalosporins / 100 resident-days.**
7. **Number of prescriptions of amoxicillin-clavulanate / 100 resident-days.**
8. **DDDs of amoxicillin-clavulanate / 100 resident-days.**
9. **Number of prescriptions of cephalosporins / 100 resident-days.**
10. **DDDs of cephalosporins / 100 resident-days.**
11. **Number of prescriptions of quinolones / 100 resident-days.**
12. **DDDs of quinolones / 100 resident-days.**
13. **Number of prescriptions of MLSK / 100 resident-days.**
14. **DDDs of MLSK / 100 resident-days.**
15. **Number of prescriptions of topical antibiotics / 100 resident-days.**
16. **Number of prescriptions of parenteral antibiotic / number of prescriptions of oral + parenteral antibiotics.**
17. **Number of prescriptions of more than 1 antibiotic on the same day / number of antibiotic prescriptions**
18. **Number of prescriptions of antibiotics with a different antibiotic prescribed the week after the first prescription / number of antibiotic prescriptions**
19. **Number of urine cultures / 100 resident-days.**
20. **Number of residents having at least 1 urine culture per year / total number of residents per year.**
21. **Number of urine cultures / total number of residents per year.**

**List of proxy indicators to evaluate the relevance of antibiotic prescriptions at nursing home level.***We suggest that participants read the scientific evidence based supporting the definition of each proxy indicators detailed in attachments No.3 and No.4.*

1. **Antibiotic prescriptions against UTI in men:** number of prescriptions of nitrofurantoin + certain quinolones (norfloxacin, enoxacin, lomefloxacin) + fosfomycin-trometamol / number of prescriptions of antibiotics for the year for male residents. **Optimal target 0 and acceptable target < 0.5.**
2. **Antibiotic prescriptions against UTI in women:** number of prescriptions of nitrofurantoin + pivmecillinam + fosfomycin-trometamol / number of prescriptions of quinolones for the year for female residents. **Target > 1.**
3. **Repeated prescription of quinolones:** number of prescriptions of quinolones among residents having been prescribed quinolones in the preceding 6 months / total number of prescriptions of quinolones. **Optimal target 0 and acceptable target < 10%.**
4. **Seasonal variation in total antibiotic prescriptions:** [number of prescriptions of antibiotic during the cold weather season (january-march and october-december) / number of prescriptions of antibiotic during the hot weather season (april-september) - 1] x 100. **Target < 20%.**
5. **Seasonal variation in quinolones prescriptions:** [number of prescriptions of quinolones during the cold weather season (january-march and october-december) / number of prescriptions of quinolones during the hot weather season (april-september) - 1] x 100. **Optimal target < 5% and acceptable target < 10%.**
6. **First-line antibiotics / second-line antibiotics prescriptions:** number of prescriptions of amoxicillin-clavulanate / number of prescriptions of quinolones + cephalosporins + MLSK. **Target > 1/**
7. **Prescriptions of not indicated antibiotics:** number of prescriptions of lomefloxacin + moxifloxacin + certain quinolones (norfloxacin, enoxacin, lomefloxacin) + telithromycin + spiramycin-metronidazole + cefaclor + cefadroxil / total number of antibiotic prescriptions. **Optimal target 0 and acceptable target < 0.5.**
8. **Estimated duration of antibiotic prescriptions > 8 days:** Number of prescriptions > 8 days for specific antibiotics (see attachment No.4) / total number of antibiotic prescriptions for these antibiotics. **Optimal target < 5% and acceptable target < 10%.**
9. **Co-prescription of antibiotic + systemic NSAIDs:** number of antibiotic + systemic NSAIDs co-prescribed on the same day / total number of antibiotic prescriptions. **Optimal target 0 and acceptable target < 5%.**
10. **Co-prescription of antibiotic + systemic corticosteroids:** number of antibiotic + systemic corticosteroids co-prescribed on the same day / total number of antibiotic prescriptions. **Optimal target 0 and acceptable target < 5%.**
11. **Estimated flu vaccine coverage:** number of flu vaccines dispensed during the second semester / number of residents staying in the NH between October and December. **Target ≥ 90%.**

**List of experts and observers composing the national committee:**

| Experts |
| --- |
| Collège de médecine générale (CMG) Dr Bernard Clary |
| Société Française de gériatrie et gérontologie (SFGG) Pr Benoit De Wazieres et Dr Matthieu Coulongeat |
| Fédération française des associations de médecins coordonnateurs d’EHPAD (FFAMCO) Dr Nathalie Maubourguet |
| Association nationale des médecins coordonnateurs et du secteur médico-social (MCOOR) Dr Gaël Durel, Dr Frédéric Maraval et Dr Odile Reynaud-Levy |
| Société de pathologie infectieuse de langue française (SPILF)  Dr Sylvain Diamantis, Dr Thibault Fraisse et Pr David Boutoille |
| Centres régionaux en antibiothérapie (CRAtb) Dr Willy Boutfol, Dr Hélène Cormier (Pays de la Loire), Dr Joël Leroy, Dr Béatrice Rosolen (Bourgogne-Franche-Comté), Dr Patricia Pavese (Auvergne-Rhône-Alpes). |
| Réseau de prévention des infections associées aux soins (RéPIAS) Dr Agnès Gaudichon (Ile-de-France) et Dr Emmanuel Piednoir (Bretagne) |
| Conseil national de l’ordre des pharmaciens (CNOP) Dr Philippe Benoît |
| Mission Spares de Santé Publique France (SPF) Dr Catherine Dumartin et Dr Amélie Jouzeau |
| Observers |
| Caisse nationale d’assurance maladie (CNAM) Pr Joël Ankri, Dr Béatrice Van Oost, Mme Garmenick Leblanc et Mme Anne-Sophie Lelong |
| Assurance maladie (DRSM)  Dr Anicet Chaslerie (Pays de la Loire), Mme Ouarda Pereira et Mme Adeline Welter (Grand Est) |
| Ministère des solidarités et de la santé (MSS) Pr Céline Pulcini |
| Direction générale de la cohésion sociale (DGCS) Dr Chantal Erault et Françoise Jay-Rayon |
| Haute autorité de santé (HAS) Dr Sabine Benoliel |
| Santé publique France (SPF)  Dr Anne Berger-Carbonne et Dr Philippe Cavalie |
| Analysts |
| Mission Primo de Santé Publique France (SPF) Dr Antoine Asquier-Khati, Dr Colin Deschanvres et Dr Gabriel Birgand |

**References:**

1. Dyar OJ, Huttner B, Schouten J, Pulcini C. What is antimicrobial stewardship? Clin Microbiol Infect. nov 2017;23(11):793‑8.

2. Kruger SZ, Bronskill SE, Jeffs L, Steinberg M, Morris AM, Bell CM. Evaluating and prioritizing antimicrobial stewardship programs for nursing homes: A modified Delphi panel. Infect Control Hosp Epidemiol. sept 2020;41(9):1028‑34.

3. Sloane PD, Tandan M, Zimmerman S. Preventive Antibiotic Use in Nursing homes: A Not Uncommon Reason for Antibiotic Overprescribing. J Am Med Dir Assoc. sept 2020;21(9):1181‑5.

4. Marquet A, Thibaut S, LePabic E, Huon JF, Ballereau F. Three years of antibiotic consumption evaluation in French nursing homes. Médecine Mal Infect. août 2015;45(8):313‑7.

5. Nicolle LE, Bentley DW, Garibaldi R, Neuhaus EG, Smith PW, SHEA Long-Term–Care Committee. Antimicrobial Use in Long-Term–Care Facilities. Infect Control Hosp Epidemiol. août 2000;21(8):537‑45.

6. Surveillance de la résistance bactérienne aux antibiotiques en soins de ville et en établissements pour personnes âgées dépendantes. Résultats préliminaires 2019. Mission Primo. Santé Publique France.

7. Simon M, Pereira O, Hulscher MEJL, Schouten J, Thilly N, Pulcini C. Quantity Metrics and Proxy Indicators to Estimate the Volume and Appropriateness of Antibiotics Prescribed in French Nursing homes: A Cross-sectional Observational Study Based on 2018 Reimbursement Data. Clin Infect Dis. 21 août 2020;ciaa1221.

8. Versporten A, Gyssens IC, Pulcini C, Monnier AA, Schouten J, Milani R, et al. Metrics to assess the quantity of antibiotic use in the outpatient setting: a systematic review followed by an international multidisciplinary consensus procedure. :8.
